# Validity and Reliability of the Self-Administered Timed Up and Go Test in Assessing Fall Risk in Community-Dwelling Older Adults

**DOI:** 10.3390/geriatrics10030062

**Published:** 2025-04-29

**Authors:** Magda Reis, Maria Teixeira, Carlota Carvão, Anabela Correia Martins

**Affiliations:** 1 Physiotherapy, Coimbra Health School, Polytechnic University of Coimbra, Rua 5 de Outubro, 3045-043 Coimbra, Portugal; a2019134037@estesc.ipc.pt (M.R.); a2020137437@estesc.ipc.pt (M.T.); a2020144593@estesc.ipc.pt (C.C.); 2 H&TRC—Health & Technology Research Center, Coimbra Health School, Polytechnic University of Coimbra, Rua 5 de Outubro, 3045-043 Coimbra, Portugal

**Keywords:** Timed Up and Go test, self-administered, reliability, validity, risk of falls

## Abstract

**Objectives:** This study aimed to evaluate the validity and reliability of the self-administered Timed Up and Go (TUG) test—a gold standard for fall risk screening—by comparing it to the traditional face-to-face assessment conducted by a physiotherapist. **Methods:** A total of 37 community-dwelling adults—mean age 61.78 ± 6.88, 73% female—who took part in fall risk screening actions in the central region of Portugal were assessed. The protocol included sociodemographic and history of falls questions, the Self-Efficacy for Exercise questionnaire, the Activities and Participation Profile Related to Mobility (PAPM), and three functional tests, namely the 10-Metre Walking Speed (10-MWS), TUG, and 30 Seconds Sit to Stand (30 s STS) tests. Within an interval of 18–24 h after the face-to-face moment, the participants were instructed to self-administer the TUG test at home. The validity and reliability of self-administered TUG test were examined using the limits of agreement, clinically acceptable limit, intra-class correlation coefficients (ICCs), paired *t*-tests, and Pearson’s coefficient correlation (r). **Results:** The limits of agreement for self-administered assessment were within the clinically acceptable limits. The average result of the face-to-face TUG test and the self-administered TUG test was 7.47 ± 2.45 and 7.57 ± 3.10 s, respectively. When comparing the two evaluations, they were strongly associated (r = 0.716, *p* < 0.001), with an excellent ICC of 0.82 (0.65–0.91), for a 95% confidence interval and significance level of 0.05 (*p* ≤ 0.05). **Conclusions:** The use of the self-administered TUG test for the screening of risk of fall, using low-cost technology, appears to be valid and reliable in community-dwelling adults aged 50 and above. Enabling older adults to perform the TUG test at home can empower them to take an active role in managing their health and ageing process, while also offering physiotherapists regular feedback for fall prevention.

## 1. Introduction

Falls and related injuries are an important public health problem worldwide. Through screening in the community, it is possible to prevent them by identifying and stratifying the risk, to establish prevention strategies [1,2]. One of these pieces of information can be the results of a functional test, which can be fundamental for the physiotherapist to establish an appropriate clinical reasoning at that moment, provide support advice, or justify the progression of an exercise plan. Consequently, the performance of a functional test by the person with or without the help of a family member/carer is potentially useful. The global population is ageing, and it is a challenge to maintain the health and well-being of older adults [3]. With the demographic trends that have emerged, and the costs associated with healthcare, it is believed that telehealth may be suitable for monitoring and supporting not only at-home rehabilitation processes for the elderly but also monitoring functional capacity and controlling risk factors that can prevent falls. Although this modality has already demonstrated its viability and acceptance by the elderly, there is still some lack of confidence in handling the technology [1,4]. A study integrated various technologies for fall monitoring and prevention, demonstrating their viability, particularly in addressing mobility and balance. These technologies achieved high levels of satisfaction among both users and healthcare professionals, supporting regular and continuous follow-up, even in remote areas with limited access to healthcare services [5].

Digital health acts in prevention, assessment, diagnosis, monitoring, and treatment integrates categories such as telehealth [6,7]. It overcomes barriers such as distance, transport, and the availability of health professionals [8,9]. Recent studies, which contained measures of functional capacity such as the Timed Up and Go test, the 30 Seconds Sit to Stand test, and the 10-Metre Walking Speed test, have shown that this format is cost-effective, as it improves users’ adherence to treatment and reduces costs inherent in the healthcare process, with high levels of satisfaction reported [6,9,10,11]. A 2023 systematic review found that telerehabilitation is as effective as face-to-face care in assessment, pain control, functionality, and health education [11]. In terms of validity and reliability, the two types of assessment can produce similar results [6].

Self-assessment and self-monitoring are components that can be part of a physiotherapy regime practised remotely. Making the user responsible for managing and treating their condition helps them achieve better results and increases adherence to treatment, including exercise [12]. Several studies have demonstrated the effectiveness of remote functional assessment. Using the TUG test, acceptable results have been demonstrated, with excellent validity and reliability [13,14,15,16]. Functional tests, rather than maximum effort tests, can be more easily accepted in a home environment [13].

The TUG is a test strongly recommended for predicting falls in community-dwelling older adults [17,18]. As well as being simple, it assesses gait and balance through a combination of standing, sitting, walking, and turning, and is a quick way of screening for the risk of falls [18,19]. Therefore, this study aimed to test the validity and reliability of the TUG test result, self-administered at home, when compared to the same administered by a physiotherapist.

## 2. Materials and Methods

### 2.1. Design, Setting, and Participants

This study reported on a validity and reliability test model approved by the Ethics Committee of the Polytechnic Institute of Coimbra (Registration no. 149 CEIPC/2023). This study was registered at ClinicalTrials.gov (Registration no. NCT06481384, on 27 June 2024). The participants were adults aged 50 or over, were living in a community, and took part in fall risk screening actions advertised in the usual places (municipalities and associations) in the centre of Portugal. The 37 participants who agreed to repeat the TUG test at home independently, in the following 18–24 h, were not given the results of the test carried out in person.

### 2.2. Outcome Measures

The outcome measures used to collect the data belong to the FallSensing screening protocol [1], to which were added questions related to the use and preference of communication technologies such as smartphones, tablets, or computers and the availability of remote healthcare, namely telephysiotherapy.

#### 2.2.1. Self-Reported Questionnaire

The self-report questionnaire included yes/no questions to characterise the sample in terms of sociodemographic and clinical data, history of falls, fear of falling, sedentary behaviours, measured by spending over 4 h seated, 5 days or more per week, and questions on education level and health self-perception.

#### 2.2.2. Self-Efficacy for Exercise

This 5-item questionnaire intended to analyse the confidence that a person has in performing exercise according to 5 different emotional states, such as feeling worried or having problems, feeling depressed, feeling tired, feeling tense, and being busy [1,20].

#### 2.2.3. Activities and Participation Profile Related to Mobility (PAPM)

PAPM is an 18-item scale to assess the difficulties an individual experiences while performing certain daily activities in their natural environment, such as social interactions and relations, education, employment, money management, social and community life, and the influence a person’s active participation has on society. It has a 5-point Likert scale from 0, meaning “no limitation or restriction”, to 4, meaning “complete limitation or restriction”. In between, 1 indicates “mild limitation or restriction”, 2 indicates “moderate limitation or restriction”, and 3 indicates “severe limitation or restriction”. Since some activities may not apply, not all activities may be rated [1,21].

#### 2.2.4. Functional Tests

Functional capacity was assessed using a set of three tests also associated with the cited protocol [2], which are the 10-Metre Walking Speed (10-MWS) test, 30 s Seconds Sit to Stand (30 s STS) test, and TUG test. These tests assess domains such as gait, balance, functional mobility, lower limb strength and fall risk [22].

#### 2.2.5. 10-Metre Walking Speed Test

The 10-MWS test was used to calculate the speed of accelerated walking, without running, timed over a 10-metre course. This can be useful in identifying those who are at risk or in need of intervention. Performing this test requires a 20 m course, with 5 m of acceleration, 5 m of deceleration, and 10 m for timed walking. Markings are made at 5, 10, 15, and 20 m and the time between 5 and 15 m is recorded. The person is instructed to walk at their fastest speed, as well as wear comfortable footwear and walking aids if necessary [1]. A value with ≤1 m/s indicates that we should start a programme to reduce the risk of falling and ≥1.42 m/s indicates a safe speed for crossing streets [2].

#### 2.2.6. 30 Seconds Sit to Stand

The 30 s STS test is a simple instrument used to assess lower limb strength and assess muscle weakness. The person is instructed to perform cycles of sitting and getting up from a chair as many times as possible for 30 s. At the end, the number of repetitions is recorded [1]. The normative levels for the number of stands depend on age and sex [2].

#### 2.2.7. Timed Up and Go Test

The TUG test evaluates dynamic balance, mobility, and strength of the lower limbs. The test begins with the person sitting in a standard chair and is instructed to walk a 3-metre course, turn around, return to the chair, and sit down as quickly as possible, without running and without the aid of the upper limbs [1]. A score of >10 s indicates which community-dwelling older adults are more likely to fall [2].

### 2.3. TUG Testing Procedure

The participants were given a new informed consent to take part in the study and were explained its purpose and procedure. Each one was measured in person by the physiotherapist (face-to-face assessment) and at home by the participant (self-administered assessment), 18 to 24 h apart.

The face-to-face assessment was carried out by a physiotherapist. It should be noted that each participant performed a single TUG test and the result was recorded in seconds to two decimal places. More details of the protocols used for the face-to-face and self-administered TUG tests are shown in Table 1.

### 2.4. Statistical Analysis

The statistical analysis was carried out using IBM SPSS (Statistical Package for the Social Sciences) software, version 29.0.1.0, for Windows. The interpretation of the statistical analysis tests was conducted on a significance level of 0.05 (*p* ≤ 0.05), with a confidence interval of 95%. Pearson’s correlation (r) was used for the analysis of correlations. In the descriptive analysis of the variables, the data were presented according to the mean and standard deviation or percentage, whichever was more appropriate. Pearson’s correlation (r) was used for the analysis of correlations with the values: 0.9–1.0 as very strong, 0.70–0.89 as strong, 0.40–0.69 as moderate, 0.10–0.39 as weak and 0.00–0.19 as negligible [24]. The data were also presented in terms of its distribution, with a Q-Q graph and T-test for ensuring normality. For the TUG test, the minimum clinically acceptable limit of 5 s was used, as previously reported [25].

Firstly, the validity between the self-administered assessment and the simultaneous face-to-face assessment was determined using paired *t*-tests. For concurrent validity between the self-administered assessment and the separate face-to-face assessment, the Bland and Altman limit of the agreement statistic was used [16,26]. Values that were within the minimum clinically acceptable difference were considered valid and acceptable [26].

The validity of the assessment carried out by the participant at home was also established by comparing it with a separate face-to-face assessment, using the mean difference of assessments, paired *t*-tests, and intra-class correlation coefficient (ICC) [13,14,16].

The strength of reliability by ICC was analysed as follows: from 0 to 0.40 as poor, from 0.40 to 0.75 as fair to moderate, and from 0.75 to 1.00 as excellent [27].

## 3. Results

Of the 37 participants, the average age was 61.78 ± 6.88 (range 50–81) years and 73% were female.

Table 2 shows the characteristics of the participants, namely sociodemographic data, characteristics related to a history of falls, health perception, level of sedentary lifestyle, access to and preference for technologies and telehealth, the total score of the self-efficacy scale for exercise, as well as the PAPM and the functional tests carried out in person. It also presents the results of the TUG test self-administered.

Table 3 represents correlations between the three functional tests carried out face-to-face and the self-administered TUG test, which correspond with Pearson’s correlation coefficient and level of significance.

### Validity

When comparing the face-to-face with the self-administered assessment, there were no significant differences between the two assessments for the TUG test (*p* > 0.05), with a mean difference (95% confidence interval (CI)) of −0.10 (−0.87 to 0.62) seconds.

**Figure 1:**
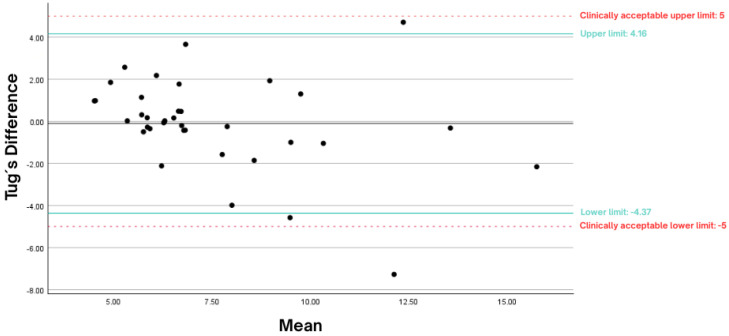
Bland and Altman plots of the difference between the mean of self-administered and separate face-to-face assessments. **Note:** Black line represents the mean difference between the two types of evaluation; green lines represent upper and lower limits; red dashlines represent clinically acceptable limits.

Figure 1 illustrates the graph with the mean difference and limits of agreement for the TUG test when comparing the assessment carried out by the participant at home with the separate face-to-face assessment.

The validity of the assessment carried out by the participant at home compared to the separate face-to-face assessment for a 95% confidence interval (95% CI) was excellent, with an ICC of 0.82 (0.65–0.91).

## 4. Discussion

To summarise, this study verified the validity, consistency, and agreement of the use of telerehabilitation, using the self-administered TUG test compared to face-to-face assessment in a group of 37 community-dwelling individuals aged 50 and over. Similar previous studies have also confirmed the validity and reliability of assessing functional capacity by telehealth using this test. The results of the present study are in line with those studies in which two types of assessment, face-to-face and remote, were compared and no statistically significant differences were found in the results of the TUG test in these two measurements (Table 3) [13,14,16].

The TUG is a test that assesses balance, gait, functional mobility, and lower limb strength [20]. When it was carried out face-to-face, it was strongly correlated with the other functional tests applied. The same was true when the test was carried out by the person themselves, which suggests that the fact that the person is responsible for carrying out this test in their home environment does not make it invalid or diminish its psychometric properties when compared to other measures.

Regarding validity, the results of this study showed no differences between the two assessments for the TUG test, confirming a high degree of concurrent validity of the self-administered functional assessment compared to the face-to-face assessment. Based on the ICC, the validity between the assessments was classified as excellent, with the limits of agreement falling within the clinically acceptable range, as shown in Figure 1. Both the upper and lower limits of the sample in this study were within the clinically acceptable range, defined as 5 s. This suggests that the self-administered TUG test demonstrated acceptable accuracy, with good consistency and a low mean difference. The results of the two assessments were closely aligned, supporting the validity of this study, as no discrepancies were found with the pre-defined limits established in the literature for this test. Therefore, these findings validate the use of this assessment method in this population, clearly indicating that a self-administered, home-based evaluation using low-cost technologies is technically feasible, valid, and reliable.

The sample in this study has an above-average functional capacity when compared to the general Portuguese population in relation to the three functional tests applied [28]. A more sedentary lifestyle is practised by more than half of the sample in this study; however, it should not be an exclusion criterion to consider when choosing a participant to obtain healthcare through telehealth, since it allowed viable results to be obtained between the two assessments.

It should be noted that most participants reported that they were willing to obtain healthcare at a distance, particularly telephysiotherapy. Similarly, the smartphone was the preferred technology. This indicates that most participants in this study are open to using technology and possess some level of digital literacy, which may have contributed to the feasibility and reliability of the method in this context. The literacy levels varied, and the overall sample included a considerable number of individuals across different educational backgrounds, demonstrating that this method can yield successful results regardless of educational level.

### 4.1. Strengths

As far as we know, this is the first study to investigate self-administered functional assessment by the user themselves in their home environment, using low-cost technology, specifically mobile phones, without the need for internet access. It uses a measure of functional capacity that is commonly used for assessment in hospitals, clinics, or other contexts, particularly in community fall risk screening, but may be more acceptable to carry out at home, bringing great benefits for empowering older adults to self-monitor their functional condition over time.

Previous studies on the validity and reliability of assessments using remote digital technology have been carried out in controlled environments with professional supervision, mostly using technologies such as videoconferencing systems as a strategy for direct communication with the user [6,10,11,13,14,15,16,29]. It is therefore questionable whether these results can be generalised to uncontrolled home environments with the person and/or carer living alone, where these types of technologies may be inaccessible, limited, or poorly handled. The sample size of this study is also considerably larger compared to most studies of this kind.

The fact that all participants took the test in person first can have a learning effect that will influence the result of the home measurement. Whilst this may seem like a bias, teaching and demonstrating the test is part of the process of empowering the person, who should never be left to their own devices when it comes to the performance of a gold standard test.

### 4.2. Limitations

There are some limitations to this study. Recruitment bias is a limitation, in that those who agreed to take part in the study may be more favourable to this assessment method, since various factors were considered, including levels of understanding and ability.

The sample exhibited high values for all functional tests, which may limit the applicability of the same method via telehealth for older adults with different characteristics.

## 5. Conclusions

Community-dwelling adults aged 50 and above can perform the TUG test independently in their own homes after receiving instructions on how to perform it, and this functional test is easy and quick to implement since this test has been successfully measured under the real-life conditions of a telephysiotherapy assessment.

Excellent validity and reliability were demonstrated between the two forms of assessment. In general, we can interpret these data when considering the context of the study design and the sample size. The limits of agreement were within the pre-defined clinically acceptable limits for this test.

Teaching older adults to perform the TUG test at home could serve as an effective strategy for remote monitoring, empowering them to manage their own health and ageing process while also enhancing digital health literacy. For physiotherapists, this method offers a valuable tool to provide periodic feedback on a patient’s current status and progress as part of a falls prevention programme.

Based on the findings of this study, this approach enables safe and remote care while effectively tracking fall risk. The method is simple, reproducible, and capable of providing an objective and quantitative assessment, with strong potential for long-term fall risk monitoring.

Nonetheless, further studies with larger samples and diverse populations are needed to generalise these findings and provide more robust evidence supporting telephysiotherapy as an effective and viable method for self-monitoring, as well as specifically exploring the results of self-administered TUG. Such studies could also help implement collaborative preventive strategies that promote active and healthy ageing while raising awareness about fall risk. To support its implementation in clinical practice, further research is required to establish more robust evidence regarding the validity of the TUG test.

## Figures and Tables

**Table 1:** Protocol used for face-to-face TUG test and for self-administered TUG test.

	Material [23]	Procedure
**TUG test** **face-to-face**	Stopwatch Standard chair 3-metre course	The physiotherapist timed how long it took for the participant to get up from the chair, walk 3 metres, and sit down again at an accelerated walking speed without running or using a walking aid.
**TUG test** **self-administered**	Leaftlet with instructions on how self-administer the test (provided by the physiotherapist) Stopwatch Standard chair 3-metre course	The physiotherapist distributed a leaflet with instructions on how to perform the TUG test at home. The instructions on how to carry out the test were the same as in the face-to-face test, with the indication of having to carry it out 18–24 h after in-person measurement. The result was returned to the researcher in seconds, to two decimal places, via SMS/phone call.

**Table 2:** Participant characteristics (n = 37).

Characteristics	
**Age (years), mean (SD)**	61.78 (6.88)
**Female, n (%)**	27 (73)
**Education, n (%)** Basic education (4–9 years) High school (10–12 years) University education	17 (45.9) 14 (37.8) 6 (16.2)
**Risk level, n (%)** Light Moderate High	18 (48.6) 13 (35.1) 6 (16.2)
**Falls in the last 12 months (yes), n (%)**	13 (35.1)
**Fear of falling (yes), n (%)**	13 (35.1)
**Health self-perception, n (%)** Very good Good Satisfactory Poor	4 (10.8) 14 (37.8) 12 (32.4) 7 (18.9)
**Sedentary behaviour (yes), n (%)**	20 (54.1)
**Access to technologies (yes), n (%)**	33 (89.2)
**Preferred technology, n (%)** Smartphone Tablet Computer	21 (56.8) 1 (2.7) 11 (29.7)
**Availability to obtain healthcare remotely (yes), n (%)**	27 (73)
**Telephysiotherapy (yes), n (%)**	21 (56.8)
**Exercise self-efficacy, mean (SD)**	14.3 (3.90)
**PAPM, mean (SD)**	0.28 (0.44)
**TUG test face-to-face, mean (SD)**	7.47 (2.45)
**TUG test self-administered, mean (SD)**	7.57 (3.10)
**10-MWS test, mean (SD)**	1.68 (0.42)
**30 s STS test, mean (SD)**	14.11(4.22)

Abbreviations: SD: Standard deviation; Exercise Self-Efficacy Scale: Total score on the Exercise Self-Efficacy Scale; PAPM: Activity and Participation Profile Related to Mobility; TUG: Timed Up and Go; 10-MWS: 10-Metre Walking Speed; 30 s STS: 30 Seconds Sit to Stand.

**Table 3:** Correlations between the TUG test (face-to-face and self-administered) and other functional tests (10-MWS and 30 s STS).

		TUG Test Face-to-Face	TUG Test Self-Administered	10-MWS	30 s STS
**TUG test face-to-face**	r	-			
	*p*				
**TUG test** **self-administered**	r	−0.716	-		
	*p*	<0.001			
**10-MWS test**	r	−0.826	−0.659	-	
	*p*	<0.001	<0.001		
**30 s STS test**	r	−0.653	−0.610	688 **	-
	*p*	<0.001	<0.001	<0.001	

**Abreviations:** r: Pearson’s correlation coefficient; *p*: Significance level; TUG: Timed Up and Go; 10-MWS: 10-Metre Walking Speed; 30 s STS: 30 Seconds Sit to Stand; ** The level of correlation is significant at 0.05 (2 ends).

## Data Availability

The data presented in this study are available on request from the corresponding author due to privacy and ethical reasons.
